# Investigating Thought Disorder in Schizophrenia: Evidence for Pathological Activation

**DOI:** 10.1371/journal.pone.0082882

**Published:** 2013-12-06

**Authors:** Ziad Safadi, Limor Lichtenstein-Vidne, Michael Dobrusin, Avishai Henik

**Affiliations:** 1 Department of Pharmacology and Physiology, University of Rochester, Rochester, New York, United States of America; 2 Department of Psychology and Zlotowski Center for Neuroscience, Ben-Gurion University of the Negev, Beer-Sheva, Israel; 3 Mental Health Center, the Faculty of Health Sciences, Ben-Gurion University of the Negev, Beer-Sheva, Israel; University of Bern, Switzerland

## Abstract

**Background:**

Previous research has yielded evidence for enhanced semantic priming in formal thought-disordered schizophrenia patients, a result that fits well with the hypothesis of disinhibited processes of spreading activation in this population.

**Objective:**

The current study examined whether hyper priming among schizophrenia patients is an outcome of further spreading of activation of a node or a result of farther activation of nodes in the semantic network. We also try to shed light on the fate of this activation.

**Methods:**

The present study tested this hypothesis by using semantic and identical priming in two different experiments. SOA (stimulus onset asynchrony) was manipulated (240 ms vs. 740 ms) within block. It is assumed that among healthy individuals, performance relies on a balance between activation and inhibition processes, contrary to in schizophrenic individuals. In order to examine this hypothesis, we compared formal thought-disordered schizophrenia patients, non thought-disordered schizophrenia patients, and healthy controls.

**Results:**

For thought-disordered schizophrenia patients, we found a large positive semantic effect and identical priming effect (129 ms and 154 ms, respectively) only with short SOA. SOA and type of priming did not modulate priming effects in the control groups.

**Conclusions:**

This result supports the claim that there is a lack of inhibitory processes among thought-disordered patients. Hyper priming in the thought-disorder group may be an outcome of hyper activation followed by rapid decay below baseline threshold.

## Introduction

One of the major positive cognitive symptoms characteristic of schizophrenia is formal thought disorder [1,2], which includes loss of abstraction ability, tangentiality, loss of word associations, derailment, thought blocking, deficits in abstract thinking and over-inclusive thinking [1,3]. Manschreck et al. [4] suggested language disorders that contribute to thought disorders could be linked with impairment of cognitive processes involved in association activation and inhibition in the semantic network. Kerns and Berenbaum [5] in their review suggested that language disorder may be related to impairment in executive functioning, increased spreading of activation, impairment of semantic processing, and impaired language production. 

These cognitive processes can be investigated using the semantic priming with lexical decision task [6] and electrophysiological measurements such as using the N400 event-related potential [7-9] and by using functional neuroimaging [10]. Semantic priming commonly produces a decrease in reaction time (RT) towards a word target that follows either a semantically related or identical prime than one that follows a semantically unrelated prime [11]. It was suggested that this effect relies on two types of processes: automatic spreading of activation (ASA) and control. The latter encompasses inhibitory processes, which allow inhibiting semantically unrelated information [12,13]. With stimulus onset asynchrony (SOA) shorter than about 250 ms between prime and target, the priming effect is thought to be the outcome of automatic spreading of activation in the semantic network. In contrast, longer SOAs give rise to processes that involve attentional capacity and strategic factors [7,14].

Neely [11] distinguished between two components of semantic priming—facilitation and inhibition. Overall, facilitation is indicated by the decrease in RT and error rates to semantically related targets compared to baseline. Inhibition is reflected by the increase of RT and error rates to unrelated targets compared to baseline. Posner [15] suggested that the facilitation effect demonstrates an automatic spreading of activation, while inhibition indicates more controlled processes as a result of the limited capacity of attention [16].

### Semantic priming and schizophrenia

Studies of semantic priming in schizophrenia have revealed contradictory results [17]. One set of studies reported strong direct semantic priming effects (hyper priming) on lexical decision latencies at short SOAs, specifically in thought-disordered schizophrenic patients [4,18,19], robust indirect semantic priming [18,20-22] and masked semantic priming [1]. Henik, Nissimov, Priel, and Umansky [23] found hyper priming in a group of chronic schizophrenics at short (240 ms) and long (1,840 ms) SOAs. Kwapil, Hegley, Chapman, and Chapman [24], using accuracy as the dependent variable, found hyper priming in schizophrenic outpatients (not selected for thought disorder), but not in bipolar patients (at 500 ms SOA). Comparing semantic priming in thought-disordered patients with normal controls, Lecardeur et al. [16] found hyper priming in those with thought disorder (TD) at both short (250 ms) and long (500 ms) SOAs.

However, various studies did not find increased semantic priming in schizophrenic patients, even when those with TD were tested separately [25-30]. In fact, significantly reduced semantic priming in schizophrenic patients has been reported, especially when SOAs longer than 500 ms were used [26,30-33]. Kreher, Holcomb, Goff and Kuperberg [34] measured event-related potentials (ERP) while subjects were performing a direct and indirect semantic task. They found an increased indirect semantic priming effect in TD relative to non-TD patients and healthy controls at 350 ms SOA conditions. At the same time, TD patients demonstrated greater indirect N400 priming effects. No direct semantic difference was found between the three groups. Spitzer [35] proposed that semantic activation should spread further in the semantic network of TD patients and result in an increased activation of semantically related information. 

Frith [36] interpreted the positive symptoms in schizophrenia as consequences of inhibition deficiency, which subsequently lead to an over activation in the semantic network. This, in turn, may generate an over awareness of the various interpretations and meanings of concepts by redundant activation of neighbor nodes [23,37-40]. 

Another explanation underlying the semantic hyper priming effect among schizophrenia patients is related to an exceptionally increased activation of associations in the semantic network [4,19,35]. Others assume that the hyper priming effect found among schizophrenia patients is due to hyper spreading of activation in the semantic network [34,41].

### Hyper priming and schizophrenia

Numerous researches were carried out to examine the source of hyper priming in semantic priming in schizophrenia. There is evidence for abnormal activation in the language brain regions among schizophrenia patients during semantic processing [42]; hence, it is still unclear what the progress of the activation of the original node (prime) is. More investigation is needed to discover whether the hyper priming is a result of faster movement of the original activation to the adjacent nodes, which means withdrawal of the activation from the original node, or whether it is a result of hyper spreading of activation farther and farther while keeping the original node at the same level of activation. The former hypothesis assumes some limited activation of nodes among TD patients, which can move faster and farther in the semantic network. The latter hypothesis (remaining hyper activation of the prime while hyper spreading of activation goes farther in the semantic network) assumes hyper resources within the semantic network of TD patients. The main goal of the current study therefore was to examine these hypotheses. For that purpose we compared identical priming and semantic priming in two separate experiments. In addition, the current study directly examined facilitation and inhibition effects by using a neutral condition as baseline. Manipulating SOAs allowed us to examine the automatic and controlled processes among patients with and without thought disorder.

## Experiment 1

### Method

#### Participants

Seventeen chronic schizophrenic patients with formal TD (4 women, 13 men), 18 chronic schizophrenic patients without thought disorder—NTD-(8 women, 10 men) and 16 normal controls (4 women, 12 men) participated in the experiment. The patients satisfied the criteria for schizophrenia as defined in the fourth Diagnostic and Statistical Manual of Mental Disorders (DSM–IV) [43]. TD was assessed according to the Brief Psychiatric Rating Scale (BPRS) [44]. Patients who scored higher than 3 out of 7 on item four of the BPRS were considered to have TD [26,41,45] (see review in [46]). Most patients had been ill for more than 9 years. The patients were selected from the Mental Health Center in Beer-Sheva, Israel. All patients continued to receive antipsychotic medication at the time of the study. The non schizophrenic controls were recruited by an ad in a local newspaper. The three groups were matched for age (for the control group, *M* = 35.8 years, *SD* = 12.6; for NTD patients, *M* = 29.7 years, *SD* = 7.3; and for TD patients, *M* = 32.8 years, *SD* = 8.4) for years of education (for the control group, *M* = 10.7 years, *SD* = 1.9; for the NTD patients, *M* = 10.8 years, *SD* = 2.1 and for TD patients *M* = 10.8 years, *SD* = 1.7) and for duration of illness (For NTD patients, *M* = 4.62 years, *SD* = 8.7 and for TD patients *M* = 7.13 years, *SD* = 10.2).

 All participants were native speakers of Hebrew. Patients had no psychiatric disorder other than schizophrenia, no history of substance abuse or dependence, and no history of severe medical or neurological disorder. All participants gave their informed consent to participate in the study. The study was carried out according to the ethical standards laid down in the 1964 Declaration of Helsinki, approved by Helsinki Committee on Human Research at Ben Gurion University of the Negev, Israel. All participants gave written informed consent via the methods approved by the Ben Gurion University of the Negev, Helsinki Ethical Committee. No minor participants were included in the study. There was no compensation for any of the subjects for participation in this study. Potential participants who declined to participate or otherwise did not participate were not disadvantaged in any way by not participating in the study. Following our study ethical approved protocol, the patient’s guardians had to give written consent as well, in case it is a requirement by patient’s medical record. According to the patients psychiatric evaluation, patients with active psychosis and those who had a compromised capacity for making decisions were excluded.

#### Stimuli

Stimuli were in Hebrew. The following examples are given in English so that the methodology is clear. Each trial consisted of a prime word and a target string of letters. There were five kinds of trials: related words (e.g., *doctor–nurse*), neutral word pairs (e.g., *xxxx–nurse*), unrelated words (e.g., *bread–nurse*), word–non-word pairs (e.g., *doctor–nruse*) and neutral–non-word pairs (e.g., *xxxx–nruse*). The unrelated pairs were created by re-pairing the primes and the targets of various related pairs. Non-words were created by scrambling the constituent letters of the target word. The non-word trials were used as fillers and were analyzed separately.

Each block consisted of 96 trials with word targets and 64 trials with non-word targets, for a total of 160 trials in each block. Every trial was composed of a prime word or xxxx string and a target. Each type of stimulus pair—related words, xxxx-word, unrelated words, xxxx*–*non-word, and word*–*non-word—was presented with equal frequency (32 trials for each condition). Half of the trials were presented using the long SOA condition (i.e., 740 ms) and half using the short SOA condition (i.e., 240 ms). Hence, for a given block, there were 5 conditions, 2 SOAs and 16 repetitions (i.e., 160 trials in block). In summary, the experiment includes two blocks (short and long SOA) for the total of 320 trials. Each block includes 20% of related condition, 20% of unrelated condition and 20% of neutral condition. Each block also included 40% of non-word targets.

#### Design

The experiment had one between-subjects and two within-subjects independent variables: group (schizophrenic with TD, schizophrenic NTD and control) was the between-subjects variable; relatedness (related, neutral and unrelated) and SOA (240 ms vs. 740 ms) were the within-subjects variables. 

#### Procedure

**Figure 1:**
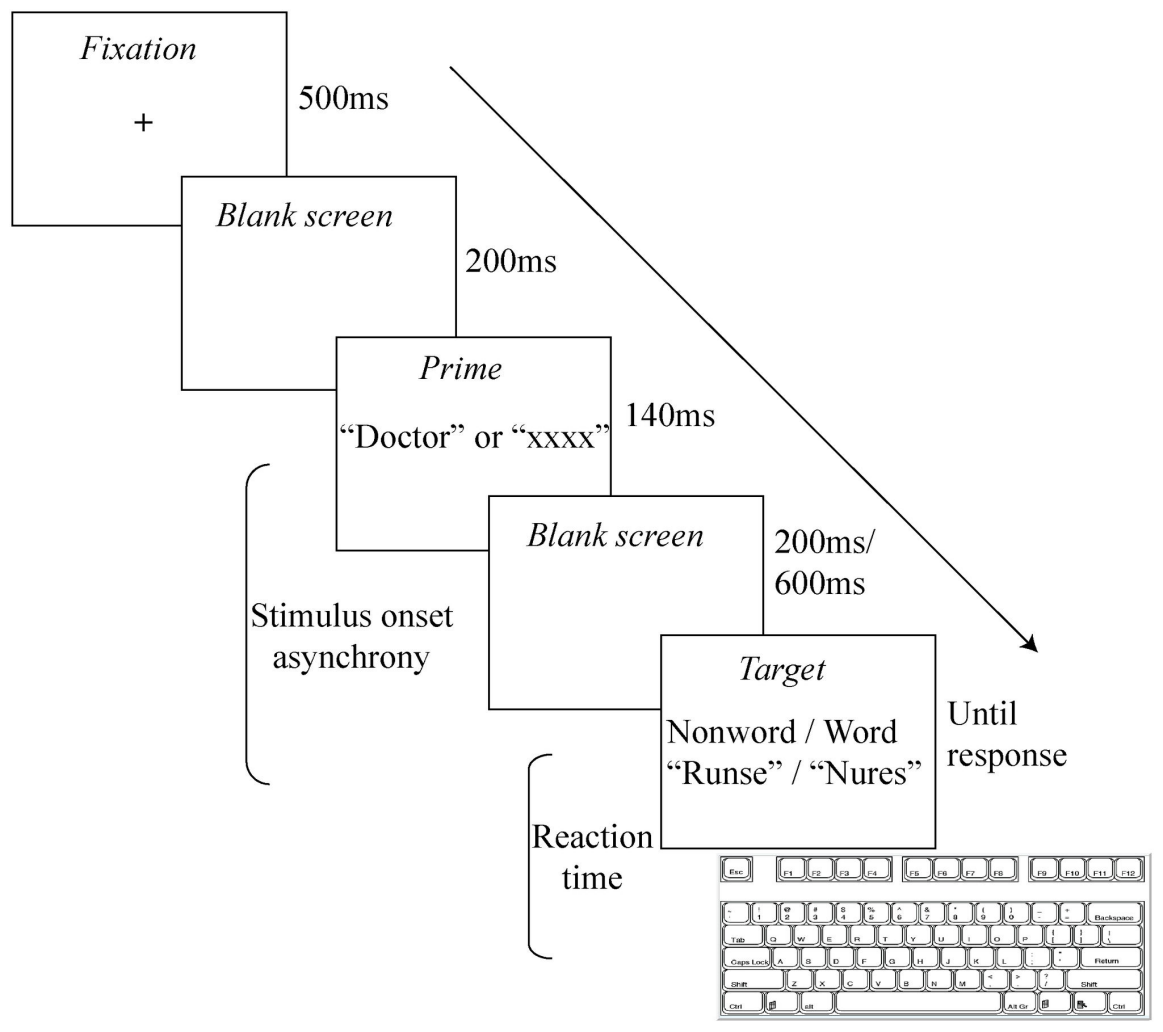
Typical sequence of events in a single trial.

A typical trial (see Figure 1) started with a fixation point for 500 ms, followed by 200 ms of a blank screen, and then a prime appeared for 140 ms. The target followed the prime after an interval of 100 ms (SOA of 240 ms) or 600 ms (SOA of 740 ms). The target exposure was terminated by the participant's keypress. Once a response was made, a blank screen was presented for 1,500 ms before another trial started. If the letter string was recognized as a Hebrew word, the participant was to press the “yes” or “no” keys as quickly as possible with the right index finger. If the letter string had no meaning, the participant was to press the “no” key as quickly as possible with the right middle finger.

#### Apparatus

A personal computer with a Windows XP operating system was used for stimulus presentation and data collection. Participants entered their lexical decisions via the computer keyboard. The participants sat 50 cm away from the computer screen. The primes and targets were presented at the center of the computer monitor. Each letter was about 4 mm wide and 4–6 mm high. 

### Results

#### Reaction time analysis

We calculated median RT for correct responses and error rate for each participant in each condition. A three-way analysis of variance (group, SOA and relatedness) showed a main effect of group, *F* (2, 48) = 10.43, MSE = 710,931, *p* < .0002. Mean RTs were 1,194 ms, 960 ms and 640 ms for TD, NTD and control groups, respectively. Additional analyses revealed that TD participants were significantly slower than NTD participants, *F* (1, 48) = 4.04, MSE = 34,124,700, *p* < .05, and controls, *F* (1, 48) = 6.96, MSE = 34,124,700, *p* < .01. Also, NTD participants were significantly slower than controls, *F* (1, 48) = 20.76, MSE = 34,124,700, *p* < .0002. The main effect for relatedness was significant, *F* (2, 96) = 18.02, MSE = 22,861, *p* < .00001. Mean RTs were 876 ms, 1,002 ms and 927 ms for related, neutral and unrelated conditions, respectively. Additionally, the two-way interaction of relatedness X group was significant, *F* (4, 96) = 2.49, MSE = 22,861, *p* < .05, as was the three-way interaction of group X SOA X relatedness, *F* (2, 96) = 18.02, MSE = 22,861, *p* < .00001. In order to reveal the source of the three-way interaction we carried out separate analyses for short and long SOAs (see Table 1).

**Table 1:** Mean, Standard Deviations and Range of Reaction Times and Error Rates for Word-Target Trials in Experiment 1.

	Short SOA			Long SOA		
	TD	NTD	CON	TD	NTD	CON
Reaction times (ms)						
Median RT						
Unrelated	1220(452)	910(249)	660(278)	1177(477)	916(293)	657(261)
	597-2297	625-1546	429-1589	630-2337	603-1717	439-1537
Neutral	1284(400)	1044(412)	673(224)	1228(409)	1108(424)	676(284)
	631-1952	713-2279	488-1327	600-2002	699-2553	423-1641
Related	1091(407)	910(285)	606(312)	1164(424)	871(300)	614(325)
	562-2072	584-1570	395-1708	548-1887	505-1705	403-1804
Priming effect (unrelated- related)	129	0	54	13	45	43
Error Rates (%)						
Unrelated	9.88	4.83	1.12	6.23	4.16	1.50
Neutral	8.29	5.77	0.75	6.94	2.22	0.75
Related	5.64	1.44	1.12	3.94	1.00	0.37
Priming effect (unrelated- related)	4.24	3.39	0	2.29	3.16	1.13

Note. TD = thought disorder patients, NTD = patients without thought disorder, CON = normal controls.

The neutral condition reaction times were slower than those of the related and unrelated conditions; therefore, we considered the trials with a neutral prime as outliers and decided not to analyze the facilitation and inhibition components of the priming effect based on reaction time. Using the words “blank” and “neutral” in the neutral condition in two different experiments replicated the same pattern of results.

#### Short SOA

The two-way interaction of relatedness X group was significant, *F* (2, 48) = 6.26, MSE = 5,852, *p* < .005. In addition, further analysis of this two-way interaction revealed two main effects of group, *F* (2, 48) = 9.48, MSE = 237,142, *p* < .0005, and relatedness, *F* (1, 48) = 16.16, MSE = 5,852, *p* < .0005. As can be seen in Table 1, the priming effect was much higher (129 ms) in the TD group than in the NTD (0 ms) and the control (54 ms) groups. Additional analysis revealed that the difference in priming between TD and the other two groups was significant, *F* (1, 48) = 9.98, MSE = 5,852, *p* < . 005. 

#### Long SOA

The two-way interaction of relatedness X group was significant, *F* (2, 48) = 9.09*,* MSE = 259,792, *p* < .0005. In addition, further analysis of this two-way interaction revealed a significant main effect for group, *F*(2, 48) = 9.45, MSE = 269,187, *p* < . 001, and relatedness, *F* (1, 48) = 21.76, MSE = 17,055, *p* < .0001. 

#### Non –Word targets

One mechanism that starts after the prime presentation is the retrospective checking on lexical decision tests. This post lexical check suggests that subjects can rely on the relationship between prime and target words to aid their lexical decisions [11]. To eliminate the post-lexical process explanation for the group differences in priming effect we have conducted a separate analysis for the non-word target trails. According to Neely [11], shorter RT for non-word probes following word prime compared to RT for non-word probes following neutral prime is an indication for the involvement of post-lexical mechanism.

Three-way analysis of variance (group, SOA and type of primes, “xxxx” vs. “word”) for the non-word trials showed significant two-way interaction between SOA and type of prime *F* (1,48)=5.16, MSE=30,937, *p* < 0.05. RTs for short SOA were 1,350 ms for word prime and 1,362 ms for neutral prime. RTs for long SOA were 1,257 ms for word prime and 1,381 ms for neutral prime. Further analysis revealed a significant main effect for prime type in long SOA condition *F* (1,48) = 4.67, MSE = 89,942, *p* < 0.05. According to Neely [14], this pattern indicates a retrospective process of semantic matching that occurs at long SOA (see Table 2). Importantly, there was no interaction with group variable, suggesting similar intact post lexical processes among schizophrenia and control groups.

**Table 2:** Mean, Standard Deviations and Range of Reaction Times and Error Rates for Non-Word-Target Trials in Experiment 2.

	Short SOA				Long SOA		
	TD	NTD	CON	TD		NTD	CON
Reaction times (ms)							
Median RT							
Prime Word	1719(878)	1250(391)	1082(785)	1489(656)		1224(442)	1058(724)
	733-3609	843-2143	571-3710	721-2885		705-2408	555-3372
Prime xxxx	1683(989)	1380(504)	1024(673)	1681(813)		1377(471)	1085(654)
	748-4718	765-2606	549-3338	731-3848		747-2522	575-3193
Error Rates (%)							
Prime Word	18	12	22	15		12	23
Prime xxxx	16	13	19	15		9	16

Note. TD = thought disorder patients, NTD = patients without thought disorder, CON = normal controls.

#### Accuracy

A three-way analysis of variance (group, SOA and relatedness) showed a main effect of SOA, *F* (1, 48) = 5.65, MSE = 130, *p* < .05. Participants made more errors (4.32%) with short SOAs than with long SOAs (3.01%). Relatedness was also significant, *F* (1, 48) = 7.96, MSE = 20, *p* < .001. Participants made 4.6%, 4.1% and 2.25% errors in unrelated, neutral and related trials, respectively. Additional analysis showed a significant priming effect (related vs. unrelated), *F* (1, 48) = 14, MSE = 20.38, *p* < .005, significant facilitation (related vs. neutral), *F* (1, 48) = 9.6, MSE = 18.44, *p* < .005, and non-significant interference. Group was also significant, *F* (2, 48) = 5.94, MSE = 147.37, *p* < .005. TD participants made more errors (6.82%) than NTD participants (3.24%) and controls (.94%). Additional analysis exhibited a significant difference between TD participants and controls, *F* (1, 48) = 11.62, MSE = 147.38, *p* < .002, and a significant difference between TD participants and NTD participants, *F*(1, 48) = 4.57, MSE = 147.38, *p* < .05.

### Discussion

Our goal in the first experiment was to examine the components of semantic hyper priming in those with thought-disorder schizophrenia. Analyzing median reaction time and accuracy showed slower processing and more error rates among TD participants compared to NTD participants and controls. The TD group showed a larger semantic priming effect at the short SOA. Our results are contradictory to those of Lecardeur et al. [16] who found hyper priming among TD participants at long but not at short SOAs. 

Likewise, Vinogradov et al.’s [30] conclusion was that the priming effect with the control groups and the hyper priming at short SOAs among TD participants demonstrate intact semantic networks. These findings are comparable to those of Manschreck et al. [4], who examined short SOAs and found hyper priming in the TD group. However, the results are inconsistent with those of Spitzer et al. [41] and Henik et al. [23], who found hyper priming in TD participants at long SOAs.

Our results also contradict those of Vinogradov et al. [30], who found a priming effect at short SOAs (250 ms) when participants performed a naming task but not when they performed a lexical decision task. 

Unpredictably, patients reacted slowly to the neutral trials. One possible explanation is that the use of "xxxx" as a non linguistic neutral stimulus was unfamiliar. Kwapil et al. [24] used the world "BLANK" as a neutral stimulus and measured the errors that subjects made instead of reaction time. They found an intermediate error rate for the neutral condition. Lecardeur et al. [16] used the word "neutral" as a neutral stimulus and found an intermediate reaction time.

## Experiment 2

In the previous experiment, a hyper priming effect was found in TD participants’ performance at short SOAs. This could be an outcome of hyper activation of the node itself or due to an enlarged spreading of activation between the nodes in the semantic network. Spitzer [35] suggested that schizophrenic patients have an abnormal activation. Our results, in addition to other evidence [47-49], suggest a distinction between node activation and activation of related nodes in the semantic network. Other studies have shown that judgment tasks such as those involving letter search or letter detection of the prime [50,51] eliminate semantic priming while repetition priming remains intact.

In order to examine the activation of the node itself, we carried out a second experiment similar to Experiment 1 except with one difference—we replaced the related condition (e.g., doctor-nurse) with an identical condition (e.g., doctor-doctor).

### Method

#### Participants

Sixteen chronic schizophrenic patients with formal thought disorder (4 women, 12 men), 17 chronic schizophrenic patients without thought disorder (5 women, 12 men) and 18 normal controls (5 women, 13 men) participated in the experiment. None of them took part in the first experiment. The participant selection and classification procedures were similar to those of Experiment 1. The three groups were matched for age (for the control group, *M* = 35.9 years, *SD* = 12.7; for NTD patients, *M* = 35 years, *SD* = 6.7; and for TD patients, *M* = 32.5 years, *SD* = 5.7), for years of education (for the control group, *M* = 10.6 years, *SD* = 2; for the NTD patients, *M* = 10.8 years, *SD* = 1.9; and for TD patients, *M* = 10.8 years, *SD* = 1.4) and for duration of illness (For NTD patients, *M* = 6.21 years, *SD* = 5.0 and for TD patients *M* = 7.14 years, *SD* = 6.93).

 All participants were native speakers of Hebrew. There were no psychiatric disorders other than paranoid schizophrenia, no history of substance abuse or dependence, and no history of severe medical or neurological disorders. All participants gave their informed consent to participate in the study. The study was performed according to the ethical standards laid down in the 1964 Declaration of Helsinki. 

#### Stimuli

The stimuli were similar to those in Experiment 1 except for one change—the related condition was replaced by an identical condition in which the prime and the target were identical words.

#### Task, procedure, design and apparatus

All these parameters were all similar to those in Experiment 1.

### Results

Similar to the first experiment, we calculated median RT and error rate for each participant in each condition. For the median RTs, we included in the analysis only trials in which responses were correct. A three-way analysis of variance (group, SOA and relatedness) showed a main effect of group, *F* (2, 48) = 10.42, MSE = 605,661, *p* < .00001. Mean RTs were 1,443 ms, 904 ms and 603 ms for TD, NTD and controls, respectively. Additional analyses revealed that TD participants were significantly slower than NTD participants, *F* (1, 48) = 23.7, MSE = 29,071,700, *p* < .00005, as well as than controls, *F* (1, 48) = 59.13, MSE = 29,071,700, *p* < .00001. Also, NTD participants were significantly slower than controls, *F* (2, 48) = 7.82, MSE = 29,071,700, *p* < .05. A main effect for relatedness was significant, *F* (2, 96) = 46.88, MSE = 14,460, *p* < .00001. Mean RTs were 893 ms, 1,051 ms and 1,006 ms for identical, neutral and unrelated trials, respectively. As well, the identical priming effect (identical vs. unrelated) was significant, *F* (1, 48) = 59.85, MSE = 10,948, *p* < .00001. None of the interactions were significant (see Table 3).

**Table 3:** Mean, Standard Deviations and Range of Reaction Times and Error Rates for Word-Target Trials in Experiment 2.

	Short SOA			Long SOA		
	TD	NTD	CON	TD	NTD	CON
Reaction times (ms)						
Median RT						
Unrelated	1438(475)	951(212)	622(204)	1449(470)	917(233)	661(166)
	727-2147	634-1351	454-1395	778-2089	611-1282	450-1196
Neutral	1538(569)	990(254)	661(212)	1521(447)	963(221)	634(188)
	843-2654	561-1351	492-1457	983-2437	579-1328	423-1315
Identical	1284(578)	809(203)	520(206)	1428(435)	795(220)	522(176)
	483-2041	499-1218	378-1312	702-2089	481-1153	381-1176
Priming effect (unrelated- related)	154	142	102	21	122	139
Error Rates (%)						
Unrelated	20.4	3.17	1.55	13.3	2.82	0.77
Neutral	9.37	6.88	1.55	10.7	3.52	2.11
Identical	9.69	3.52	0.0	17.6	3.35	1.22
Priming effect (unrelated- related)	10.7	-0.35	1.55	-4.3	-0.53	-0.45

Note. TD = thought disorder patients, NTD = patients without thought disorder, CON = normal controls.

#### Accuracy

A three-way analysis of variance (group, SOA and relatedness) showed a main effect of group, *F* (2, 48) = 10.5, MSE = 1,646, *p* < .00001. TD patients made more errors (13.5%) than did NTD patients (3.9%), *F* (1, 48) = 27.9, MSE = 1,646, *p* < .00001, and controls (1.2%), *F* (1, 48) = 146.6, MSE = 1,646, *p* < .00001. The difference between NTD patients and controls was not significant. The two-way interaction of group X relatedness was significant, *F* (4, 96) = 7.6*,* MSE = 24.5, *p* < .00005, as well as the two-way interaction of SOA X relatedness, *F*(4, 96) = 7.7, MSE = 24.5, *p* < .00005. In addition, the three-way interaction of SOA X relatedness X group was significant, *F* (4, 96) = 8.16*,* MSE = 18.6, *p* < .00005. In order to understand the source of the interaction, we carried out separate analyses for short and long SOAs.

#### Short SOA

The two-way interaction of relatedness X group was significant*, F* (2, 48) = 12.29, MSE = 23.51, *p* < .00005. Further analysis of this interaction revealed a main effect of group, *F* (2, 48) = 18.71, MSE = 102.8, *p* < .00001, and relatedness, *F* (1, 48) = 17.2, MSE = 23.5, *p* < .0005. As seen in Table 2, a repetition hyper priming effect was found among the TD group (10.75%) compared to the NTD group (0.35%) and controls (1.55%). Additional analysis show a significant effect increase in the TD group compared to controls, *F* (1, 48) = 15.2, MSE = 23.51, *p* < .0005, and to the NTD group, *F* (1, 48) = 21.6, MSE = 23.51, *p* < .00005. Analyzing the interference effect (unrelated vs. neutral) demonstrated two main effects of group, *F* (2, 48) = 17.8, MSE = 29.9, *p* < .00001, and relatedness, *F* (1, 48) = 6.25*,* MSE = 28.8, *p* < .00001. The two-way interaction of group X relatedness was also significant, *F* (2, 48) = 16.8, MSE = 28.8, *p* < .00001. Significantly larger interference was found in the TD group (11.1%) compared to the NTD group (-3.71%), *F* (1, 48) = 31.8*,* MSE = 28.3, *p* < .00001, and controls (0.55%), F (1,48) = 16.5, MSE = 28.3, *p* < .0005. The different between the NTD and control group was not significant. 

#### Long SOA

Similar to the short SOA, we analyzed separately the priming effect (identical vs. unrelated), the facilitation, and the inhibition for the three groups. Analyzing the identical priming demonstrated a group main effect, *F* (2, 48) = 34.0, MSE = 29.3, *p* < .00001. No significant facilitation was found. Analyzing the inhibition component showed a group main effect, *F* (2, 48) = 27.9, MSE = 38.0, *p* < .00001.

#### Specific comparisons

In order to gain a better understanding of the differences between the mental processes in each group, we compared the performance of every group in the two experiments. However, it seems that due to the special design of the two experiments and the nature of the groups, the observed power for this four-ways analysis of variance was impaired (1-β < 0.2). Therefore, we carried out two specific comparisons that we believe have noteworthy theoretical value. 

In the first comparison, we found a significant simple effect of experiment (type of priming) among the control group *F* (1, 32) = 11.44, MSE = 87,037, *p* < 0.01, (the means of RTs for semantic priming and for identical priming were 49 ms and 121 ms, respectively).The second comparison revealed a significant effect of SOA among TD group *F* (1, 31) = 5.73, MSE = 252,532, *p* < 0.05, but not among the other two groups. The priming effect found larger at short SOA 141 ms compared to long 17 ms.

### Discussion

Our goal in the second experiment was to investigate the source of the hyper priming found in the first experiment—whether it was due to hyper activation of the node or due to spreading of activation in the semantic network while the activation of the node itself remains normal. 

The identical priming effect that was found for both patient groups suggests that the semantic network in the TD patients was normal. The lack of any significant interaction between the priming effect and SOA suggests a similar pattern of RTs in different association conditions in the three groups. This result implies that there is a special facilitation effect within the schizophrenic participants. These results are in accord with the claim of Manschreck et al. [4], suggesting the activation (found in Experiment 1) is a result of an enhanced activation of the associations and not of the node itself. 

Analyzing the error rates shows an inhibition effect in patients with thought disorder at the short SOA trials but not at long SOA ones. This result implies an automatic inhibition process, that is, the inability of the patients to divert their attention from the stimulating node (not connected to the priming stimulus) to the target at short SOAs. 

Unfortunately, we were not able to execute a comparison between all the variables in the two experiments due to lack of statistical power in the four-way analysis of variance. However, in light of the high complexity in this study due to the participants' characteristics and experimental design the lack of power was quite anticipated. Importantly, in the specific comparison we found that the control group improved its performance in the identical priming experiment compared to the semantic related priming. However, the schizophrenia group failed to gain the same benefit. A different comparison showed that only the TD group demonstrated hyper priming in the short SOA, which is known as an indication for ASA processes. On the other hand, the NTD and control groups showed a similar priming effect in both short and long SOA, indicating both strategic along with ASA processes. 

## General Discussion

The current study was carried out to: a) Examine the inhibition and facilitation components among schizophrenics with and without formal thought disorder. b) Replicate previous contradicting findings of hyper priming. c) Examine the abnormal activation among schizophrenia patients to discover whether it is a result of abnormal spreading of activation or abnormal activation of the node itself. 

According to the findings in this study, we suggest that: Hyper semantic priming in automatic process (at short SOAs) in the TD group.

1 No priming (semantic, not identical) effects were found in the TD group at long SOAs.
2 No benefit from using identical pairs instead of semantic related pairs in the TD participants compared to the NTD and normal participants.
3 Post lexical processes were similar for controls and patients.
4 Activation of the node in patients (with and without TD) was similar to normal activation processes. Identity priming was similar across all groups.

According to our results, the enhanced priming effect appears in a rather stable way during short SOAs compared to long SOAs. This pattern of results is an indication for control process rather than automatic process in the semantic network [12,13]. Moreover, it seems that the activation of the node itself is similar to the one that appears in the control participants (Experiment 2). Therefore, the impression is that the enhanced activation that appears in the NTD patients in the long SOA conditions is connected to an enhanced activation spread [35], or to an enhanced activation of the associations [4]. 

TD patients demonstrated hyper semantic priming at short SOAs (Experiment 1). Compared to NTD and normal participants, they did not gain any benefit from exchanging semantic pairs with identical pairs. This implies hyper spreading of activation rather than hyper activation of the node itself. Furthermore, the difference in the performance of the TD participants under short vs. long SOAs explicitly revealed rapid withdrawing of the activation from the node. This withdrawing of activation may provide insight into the formal thought disorder found in this group, such as whether it entails tangential or flight thoughts. 

Importantly, one can argue that the smaller effect among the control group is due to their faster reaction time. However, we found no relation between RTs and priming effect. For instance, in experiment 1, TD group were comparable in the short and long SOA conditions. In contrast, the relative priming effects were highly different (129 ms vs. 13 ms, respectively). Moreover, control group has comparable RTs in experiment 1 and 2 but shorter than TD, yet the control group priming effects were 43 ms and 139 ms. Hence, it seems that the smaller priming effect can’t be explained by faster performance.

A facilitation effect appeared in conditions that contained non-word targets. This result implies compatibility in the semantic retrospective mechanism in regard to long SOA conditions. This finding is in accord with Neely [14]. Nevertheless, no interaction effect was found with the group type factor, suggesting that even within the schizophrenic patients the post lexical mechanism was well formed. This contradicts the conclusions of Vinogradov et al. [30], who claimed that post lexical mechanisms are defective in this population. 

Another point relates to the fact that despite a variety of studies that were conducted on schizophrenic patients, most of the studies (presented in the Introduction section) were *meaning proximity* studies that used lexical decision tasks. The popular approach is that the enhanced meaning proximity effect among schizophrenic participants is due to malfunctioning in the control and management systems, and actually reflects the difficulty in inhibition processes [23,36-40]. Other views claim that the central problem of schizophrenics does not revolve around difficulties in inhibitive processes, but rather around strong activation of the associations [4,41]. Also, there is a different approach that suggests the focus should be on the difficulty of the patients to allocate attention resources adequately [23]. The hyper priming found in positive schizophrenia may be due to the increased level of dopamine [52] as well the hyper spread of activation as a result of malfunction of the local GABAergic inhibitory circuit responsible for neuronal selectivity tuning [53].

In sum, it can be concluded that the enhanced semantic proximity effect found among schizophrenics with TD is a result of enhanced association activation. This large effect does not seem to originate from a malfunction in the post lexical inhibitory processes or enhanced activation of the node itself. The semantic meaning proximity that was sustained among all groups strongly suggests that the semantic network of the schizophrenic patients was normal [30]. The hyper semantic priming but not identical priming found at short SOA and which failed to show at long SOA among the TD group can be explained by the activation-maintenance model [54]. When the prime is presented, semantic disinhibition leads to stronger spread of activation in the network; semantically related concepts are excited automatically and activation spreads quickly as a result of relatedness. Immediately after the activation, the node decays back below threshold [3].
